# Leveraging polymerase chain reaction technique (GeneXpert) to upscaling testing capacity for SARS-CoV-2 (COVID-19) in Nigeria: a game changer

**DOI:** 10.11604/pamj.2020.35.2.22693

**Published:** 2020-04-21

**Authors:** Olanrewaju Oladimeji, Bamidele Paul Atiba, Daniel Adedayo Adeyinka

**Affiliations:** 1 Center for Community Healthcare, Research and Development, Nigeria; 2 Department of Public Health, Walter Sisulu University, Eastern Cape, South Africa; 3 Faculty of Health Sciences, Durban University of Technology, South Africa; 4 Federal Teaching Hospital, Ido-Ekiti, Ekiti State, Nigeria; 5 Department of Public Health, Federal Ministry of Health, Abuja, Nigeria; 6 Department of Community Health and Epidemiology, College of Medicine, University of Saskatchewan, Saskatoon, SK, S7N 5E5, Canada

**Keywords:** Polymerase chain reaction, GeneXpert, SARS-CoV-2, COVID-19, Nigeria

## To the editors of Pan African Medical Journal

Nigeria has confirmed 288 cases of coronavirus disease, caused by severe acute respiratory syndrome coronavirus-2 (SARS-CoV-2) infections, with seven deaths. As at 9th April 2020, about 5,000 people have been tested [1]. Nigeria currently has nine testing centres: three in Lagos, two in the Federal Capital Territory and one each in Ibadan (Oyo State), Ede (Osun State), Irruah (Edo State) and Abakaliki (Ebonyi State). Testing centres in Maiduguri, Kano, Kaduna, Jos, Sokoto and Port Harcourt are to be activated soonest [2]. Compared to other countries, there is slow infectivity rate of the virus, which is general believed to be a “tip of the iceberg” of the disease rather than the true representation. With over 9,000 people to be traced and tested, a reasonably higher number of daily tests is required to reduce the community transmission of the virus. The polymerase chain reaction (PCR) based GeneXpert for tuberculosis (TB) diagnosis may be the breakthrough and indeed a game changer to rapidly scaling up the testing capacity. It has additional advantages of more than 95% specificity and sensitivity and by extension very high reliability, being a PCR-based technique [3].

**Figure 1:**
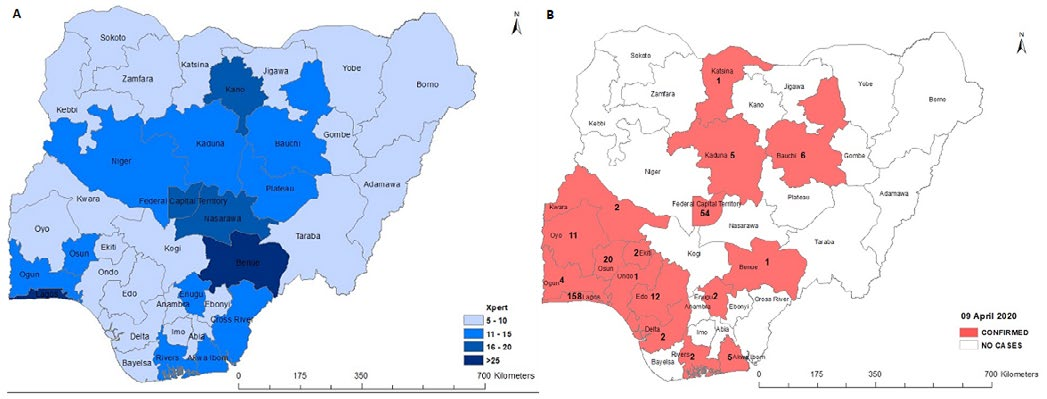
(A) coverage of GeneXpert machines in Nigeria; (B) map showing confirmed cased of COVID-19 in Nigeria as at 9^th^ April 2020

GeneXpert is a cartridge-based nucleic acid amplification machine that makes use of the polymerase chain reaction technique to test for TB and the rifampicin-resistant TB. The technology was endorsed by the World Health Organization (WHO) in late 2010 and its implementation has revolutionized the management of tuberculosis globally [3]. What is required is a new assay and cartridge, specifically targeted at the SARS-CoV-2 ribonucleic acid (RNA) and Cepheid, the manufacturer of the machine has commenced the production of “Xpert Express SARS-CoV-2” test cartridges [4]. Nigeria has extensive coverage of GeneXpert machines across the thirty-six states and the Federal Capital Territory (Figure 1 A). Regional distribution showed that the North Central has 101 (25%) of the machines, South West, 83 (21%); North West, 66 (17%); South South, 62 (15%) while North East and South East have 66 (11%) each. The Federal Government of Nigeria needs a swift move and maximize this window of opportunity to actively partner with the main TB stakeholders in Nigeria to utilize some of the 398 GeneXpert machines to test for COVID-19 and this will indeed be a quick win to solve the challenges of testing coverage [5]. It is interesting to note that Lagos State has highest number of COVID-19 (158 cases) and GeneXpert machines (34 machines) among all the states. The Federal Capital Territory, Abuja with 54 COVID-19 cases has 17 GeneXpert machines, Osun and Oyo are contiguous states in the South West zone of the country with 31 cases of COVID-19 altogether have 22 machines. Edo State has 12 cases with 8 machines and Bauchi has 8 cases with 12 machines [3] (Figure 1 B).

The Xpert Express SARS-CoV-2 test would rapidly increase the testing capacity of Nigeria. The machine can yield test result within 45 minutes and it requires a few minutes of hands on time to prepare the samples. The module 4 GeneXpert machines are the ones that are widely available in Nigeria; this module has the ability to handle four samples per cycle. Therefore, the large number of available machines throughout Nigeria will help to run hundreds of samples and can be quadrupled if more machines are procured. Early case detection will improve the epidemiological outlook of COVID-19 in Nigeria by reducing the community transmission and ultimately flattening the curve. The Nigeria Centre for Disease Control (NCDC) should endeavor to be accurate about the number of people tested daily to douse the palpable fear of the Nigerian citizens. The recent increase in detected cases points to ongoing community transmission. Therefore, as testing capacity is being increased with this innovative step of using the GeneXpert machine, government may need to consider extending the density control measures, especially in Lagos and Abuja, Osun and also work closely in unity with Oyo state government to be pragmatic about implementing similar density control measures. The expanded testing capacity may create access for prisoners and internally displaced persons to be tested for COVID-19. Outbreak among these groups of most vulnerable population may be a national disaster. More importantly, government and stakeholders need to procure more GeneXpert machines, especially the bigger modules to maximize the capacity to test. The NCDC, National Tuberculosis and Leprosy Control Programme (NTBLCP), WHO and United States Agency for International Development (USAID) as a matter of necessity should meticulously manage the “machine-shift” to prevent possible neglect to diagnosing TB and multi-drug resistant TB in Nigeria.

## Competing interests

All authors declare no competing interests.

## References

[1] Nigeria Centre for Disease Control Coronavirus (Covid-19) Highlights.

[2] Africanews Coronavirus-Nigeria: The Lagos State Biosafety Level-3 Laboratory activated for COVID-19 testing..

[3] Tbfacts.org Information about tuberculosis. Genexpert.

[4] Ike CK Securing the new COVID-19 Test Kit for Nigeria (2).

[5] The Nation FG to convert over 300 Gene Xpert TB machines for COVID-19.

